# Book Review

**Published:** 1904-09

**Authors:** 


**The Physiognomy of Mental Diseases and Degeneracy.** By JAMES SHAW, M.D. Pp. xii., 83. Bristol: John Wright & Co. 1903.—Dr. James Shaw has done well to republish in book form, with some amount of revision, the interesting articles by him which appeared in various years in the *Medical Annual.* To be able to make a diagnosis—tentative or absolute—of the various forms of mental derangement is a desirable acquirement, and one which can be facilitated by a study of this work. With some help from other alienist friends, Dr. Shaw has brought together in the text a valuable body of notes upon the appearance, behaviour and physical characteristics of the different types of the insane and mentally degenerate. The concise and clear language in which the phenomena are described make the observations exceptionally valuable. Chapter xii., on The Stigmata of Degeneration, as the indications of degeneration are known, is particularly good and illustrated with many helpful pictures. The book is indeed abundantly illustrated throughout, but we confess that many of the pictures do not seem to carry conviction with them, for example, Fig. 11, representing Delirium Tremens. This in the nature of things is perhaps inevitable; nothing short of a cinematograph could adequately reproduce the physiognomy and gestures associated with this mental state, whose chief characteristic is an ever changing mobility. On the other hand, of such mental conditions as are associated with corporeal defects or malformations—such as microcephalic idiocy, cretinism and so forth—the illustrations are admirable and have much verisimilitude. This interesting little book proves that Shakespeare spoke with less than his usual accuracy of observation when he said—

"There 's no art
To find the mind's construction in the face."

**Transactions of the Association of American Physicians.** Vol. XVIII. Pp. xxiii., 716. Philadelphia. 1903.—We are accustomed to look for the evidences of good and original work in these *Transactions*, and this volume seems to us to be of more than usual importance from the number of valuable contributions to medical science that it contains. We fear that in *Transactions* generally there is published an amount of good work that fails to receive adequate attention. Without making invidious distinctions, and with the proviso that all the papers in this volume will repay perusal, we would draw the reader's attention to the several contributions dealing with tuberculosis, especially from the experimental side, to the paper by Dr. McCallum on The Production of Specific Cytolytic Sera for Thyroid and Parathyroid, to Dr. Crozer Griffith's article on Thymus Death, to Dr. Wm. Osler's paper on Chronic Cyanosis with Polycythæmia and Enlarged Spleen, to articles on Splenic Anæmia by Drs. Dock and Warthin and by Dr. Morse. There is also an important contribution by Dr. Lewellys Barker on The Pathological Changes in the Nervous System in two of Dr. Sanger

Brown's celebrated cases of Hereditary Ataxia, and an exhaustive paper by Dr. C. L. Dana on Acute Bulbar Paralysis. Articles by Dr. H. A. Hare and Dr. Cabot respectively on The Action of Alcohol in Disease will also be found of interest; and in conclusion we can only advise our readers to give the whole volume a careful study, and commend the provision of an index.

**Transactions of the Epidemiological Society of London.** Vol. XXII., 1902–1903. London: Williams & Norgate. 1903. —The papers included in this volume are of exceptional interest and great practical importance. We would especially call attention to Professor Delépine's papers on Epidemic Diarrhœa and Food Poisoning, and on the addition of preservatives to milk. It cannot be too strongly impressed upon the medical profession that zymotic diarrhœa is a form of food poisoning, and one which can be prevented by proper attention to the storage and preparation of milk. A paper by Dr. Louis Parkes on The Prevention of Diphtheria Outbreaks in Hospitals for Children is full of practical hints as to how such epidemics may be prevented. Incidentally he mentions that 1.9 per cent. of children admitted with pseudo-diphtheria bacilli in the nose and throat subsequently developed clinical diphtheria; but as 1.6 per cent. of cases showing no pathogenic bacilli also developed the disease, it will be seen that the detection of the pseudo-bacillus is of small importance. One of the most interesting articles is by Jonathan Hutchinson on The Etiology of Leprosy, in which he upholds with characteristic skill the theory that the specific cause gains access to the system by the stomach in the eating of fish which is in a state of commencing decomposition.

**A Non-Surgical Treatise on Diseases of the Prostate Gland and Adnexa.** By George Whitfield Overall, M.D. Pp. 207. Chicago: Rowe Publishing Company. 1903.—This book is founded on considerable knowledge of the pathology of prostatic and urethral infections, and of scientific methods for their treatment. It is prefaced by the remarks, "Damage once done to the prostate by the knife is irreparable. Better bear the ills we have than fly to those we know not of." The author is particularly critical upon Bottini's operation, and we think is justified in denouncing its general use. He describes in full what he calls his own method of treating the enlarged prostate at the neck of the bladder by repeated slight cauterisations with a special instrument, or by electrical cataphoresis. He says, "After having allayed the acute symptoms, I 'hammer' at the prostate both through the rectum and urethra until the indurated tissue begins to soften, then atrophy." Speaking of neurasthenia, he says, "The disease so commonly referred to as 'nervous prostration' might, in the large majority of instances, be traced to the prostate, should the attending physician take the care to examine the patient for this trouble." We can appreciate his point of view from the standpoint that the prostate is a "sexual brain," although it is the first time we have read of a man's wits on his beam ends. Those interested in this branch of treatment may gleam some useful hints from this book, although we cannot endorse some statements, and others seem a bit "tall."

**Cancer of the Uterus.** By Arthur H. N. Lewers, M.D. Lond., F.R.C.P. Lond. Pp. vii., 328. London: H. K. Lewis. 1902.—The chief object of the author appears to be to emphasise the good results which can be obtained by early operation in cases of uterine cancer. This point is exemplified by nineteen cases that have lived some years since operation, but as they form only about 2 or 3 per cent. of the total number of cases of cancer of the uterus seen by the author it is clear that the disease in question leaves much to be desired in general results. The fact that so many cases come too late for any radical operation to be successfully performed is attributed to the ignorance of women of the early symptoms of cancer, and to obviate this a suggestion is made that a leaflet should be issued by some impersonal authority to doctors, matrons, and nurses, setting forth the importance of recognising suspicious signs occurring about the menopause. An unnecessary amount of space is taken up in the description of individual cases, but the varieties, symptoms and treatment of the different forms of malignant growths are clearly described. In the account of "pan-hysterectomy" no mention is made of the removal of glands, which would appear desirable if the removal of the uterus for cancer is to be brought into line with operations for cancer in other parts of the body. Deciduoma malignum receives only an incidental mention, but it is of sufficient importance to have had a chapter devoted to its pathology, symptoms and treatment. The book is freely illustrated by serviceable woodcuts and coloured plates.

**The Manual Treatment of Diseases of Women.** By Gustaf Norström, M.D. Pp. 230. New York: G. E. Stechert. 1903.—This monograph is based on observations gathered from an experience in pelvic massage extending over more than twenty-five years. An account is given of the history of this method of treatment, and the various objections which have been urged against it are discussed. The author appears to have had considerable success in the treatment of all chronic inflammatory affections of the uterus and appendages, and the histories of a large number of cases are given. In the treatment of uterine displacements by pelvic massage it is not claimed that the abnormal position of the uterus is rectified, but that the chronic endometritis and metritis (which are the cause of the pain) are cured. The treatment is recommended as a palliative and harmless measure in the case of small fibroids, giving rise to menorrhagia, metrorrhagia, and leucorrhœal discharge; there does not appear to have been any marked diminution in the size of the tumours in the cases recorded, although the menorrhagia and other symptoms were relieved. In Chapter xiv., which deals with diseased conditions of the tubes and ovaries, the author very rightly advises great caution in the application of the treatment, on account of the danger of rupturing a distended tube, and thus setting up peritonitis. Although in some cases of chronic metritis and parametritis this method of treatment seems to be beneficial, we do not think it will ever be largely employed in this country.

**Diseases and Injuries of the Eye.** By GEORGE LAWSON. Sixth Edition. Revised and in a great measure rewritten by ARNOLD LAWSON. Pp. xx., 587. London: Smith, Elder & Co. 1903.—It is now nearly twenty years since the appearance of the Fifth Edition of George Lawson's text-book in "Renshaw's Manual" Series, and those who are familiar with that admirable little book will cordially welcome this new and much enlarged edition, revised, and in a great measure rewritten, by Arnold Lawson. The general arrangement of the work has been entirely altered, and many chapters, such as those on elementary optics and the development of the eye, have been added so as to make the work complete. At the same time its practical utility as a clinical handbook, the distinguishing feature of previous editions, has been retained. The language is clear and definite, and the reviser has not hesitated to relate his individual experience. By most praiseworthy and exhaustive bacteriological examination of more than two hundred healthy conjunctival sacs, he is able to disprove the statements of authors which have led to the conjunctival sac being regarded as a "species of anatomical sink, teeming with all manner of micro-organisms." His facts go to show that the conjunctival sac and its secretion exert a detrimental influence on the growth of bacteria. Of course, omissions when space is limited are inevitable, but we should have expected some reference to the use of adrenalin, and the therapeutic use of X-rays. On the whole, however, the work is singularly accurate and up to date, and is furnished with a concise table of contents, a full index, and a formulary of prescriptions. These features, and especially the thoroughly practical character of the work, render it particularly well fitted to meet the requirements of the busy practitioner.

**Eye Symptoms as Aids in Diagnosis.** By EDWARD MAGENNIS, M.D. Pp. 108. Bristol: John Wright & Co. 1903.—Without knowing more of the peculiar requirements of Irish Poor Law medical officers—for whom this book is specially intended—we cannot say how far it supplies a real want. It covers, or rather extends over, a very wide field, and undoubtedly contains much information, but it is frequently ambiguous, inaccurate, or misleading. The spelling and punctuation are by no means faultless. We are told that "a bilateral cataract" may develop during the course of diabetes, and that nystagmus (defined as "a rapid oscillating movement of the eyes in one direction") is "not of any special diagnostic moment to the physician," whilst inequality of the pupils is pathognomonic of general paralysis. Twelve pages are devoted to "some ophthalmic terms and their meanings," and contain a fund of "latinity" (ranging from *rhytidosis corneæ* to *lapides cancrorum*) which should satisfy the requirements of any practitioner.

**Lectures on Massage and Electricity in the Treatment of Disease.** By THOMAS STRETCH DOWSE, M.D. Fourth and Revised Edition. Pp. viii., 454. Bristol: J. Wright & Co. 1903.—This book has already been reviewed in our columns, and, as the volume before us is the fourth and revised edition, it is hardly necessary to say more than that it presents the same features as former editions, with the additions mentioned below. When a work has reached the fourth edition it has evidently gained an audience to whom it is useful, and is too well known to be affected by praise or blame from the reviewer. No one would now dispute the author's statement in his preface that "massage . . . is an important physical aid in the treatment of diseased states." The same statement can be made with equal safety with regard to electricity. There is no doubt that every medical man should have some acquaintance with the applications of massage and electricity, should know what can and what cannot be done by them in the alleviation of disease, and be prepared to use both in an intelligent way. If this desirable consummation were reached we should hear less of the abuses of these forms of treatment in unqualified hands. To the section on electro-physics the author has added an appendix dealing with the X-rays, with high-frequency currents and with phototherapy. The mode of production and apparatus necessary for the X-rays and high-frequency currents are described, and directions given for their practical application in medical treatment.

**A Manual of Ambulance.** By J. SCOTT RIDDELL, M.B., M.A. Pp. xvi., 227. London: Charles Griffin & Co., Ltd. 1904.—This, the fifth edition of a useful little work on "first aid," is now brought up to date. It is well illustrated, and the subject matter is placed before the reader in a simple manner. The examination papers at the end of the volume are of the character that is required for those wishing to know something of "first aid"; they are not too deep, and the information asked for in them does not, as so often happens, lead the learner to think that he can do without surgical aid from a medical man.

